# 3D volume reconstruction from serial breast specimen radiographs for mapping between histology and 3D whole specimen imaging

**DOI:** 10.1002/mp.12077

**Published:** 2017-03-17

**Authors:** Thomy Mertzanidou, John H. Hipwell, Sara Reis, David J. Hawkes, Babak Ehteshami Bejnordi, Mehmet Dalmis, Suzan Vreemann, Bram Platel, Jeroen van der Laak, Nico Karssemeijer, Meyke Hermsen, Peter Bult, Ritse Mann

**Affiliations:** ^1^ Centre for Medical Image Computing University College London WC1E 6BT London UK; ^2^ Diagnostic Image Analysis Group Radboud University Medical Center 6500 HB Nijmegen The Netherlands; ^3^ Department of Pathology Radboud University Medical Center 6500 HB Nijmegen The Netherlands; ^4^ Department of Radiology Radboud University Medical Center 6500 HB Nijmegen The Netherlands

**Keywords:** 3D volume reconstruction, breast histology‐radiology registration, serial breast specimen images

## Abstract

**Purpose:**

In breast imaging, radiological in vivo images, such as x‐ray mammography and magnetic resonance imaging (MRI), are used for tumor detection, diagnosis, and size determination. After excision, the specimen is typically sliced into slabs and a small subset is sampled. Histopathological imaging of the stained samples is used as the gold standard for characterization of the tumor microenvironment. A 3D volume reconstruction of the whole specimen from the 2D slabs could facilitate bridging the gap between histology and in vivo radiological imaging. This task is challenging, however, due to the large deformation that the breast tissue undergoes after surgery and the significant undersampling of the specimen obtained in histology. In this work, we present a method to reconstruct a coherent 3D volume from 2D digital radiographs of the specimen slabs.

**Methods:**

To reconstruct a 3D breast specimen volume, we propose the use of multiple target neighboring slices, when deforming each 2D slab radiograph in the volume, rather than performing pairwise registrations. The algorithm combines neighborhood slice information with free‐form deformations, which enables a flexible, nonlinear deformation to be computed subject to the constraint that a coherent 3D volume is obtained. The neighborhood information provides adequate constraints, without the need for any additional regularization terms.

**Results:**

The volume reconstruction algorithm is validated on clinical mastectomy samples using a quantitative assessment of the volume reconstruction smoothness and a comparison with a whole specimen 3D image acquired for validation before slicing. Additionally, a target registration error of 5 mm (comparable to the specimen slab thickness of 4 mm) was obtained for five cases. The error was computed using manual annotations from four observers as gold standard, with interobserver variability of 3.4 mm. Finally, we illustrate how the reconstructed volumes can be used to map histology images to a 3D specimen image of the whole sample (either MRI or CT).

**Conclusions:**

Qualitative and quantitative assessment has illustrated the benefit of using our proposed methodology to reconstruct a coherent specimen volume from serial slab radiographs. To our knowledge, this is the first method that has been applied to clinical breast cases, with the goal of reconstructing a whole specimen sample. The algorithm can be used as part of the pipeline of mapping histology images to ex vivo and ultimately in vivo radiological images of the breast.

## 1 Introduction

Histopathological imaging is currently used as the gold standard for characterizing the tumor microenvironment and estimating resection margins following surgery, while radiological imaging is typically used for diagnosis, therapy monitoring, and image‐guided interventions. Relating the information available across scales could lead to a better understanding of the information available in the in vivo radiological imaging and the in vivo image signal modulation in terms of the underlying tissue microstructure. This in turn has the potential to enhance in vivo tumor characterization, thereby improving therapeutic decision‐making and, ultimately, patient prognosis and treatment outcomes. Mapping specific tumor microenvironment biomarkers (such as hypoxia, proliferation, and increased blood flow) back to the preoperative imaging provides a means of validating imaging biomarkers.^1^ It can also be used as a tool to validate current tumor segmentation protocols and methods used routinely in radiation therapy planning and image‐guided interventions, ensuring that all diseased tissue is treated, while sparing as much healthy tissue as possible.

Accurate alignment of the images across scale can be problematic, however, due to the large deformation and potentially physiological and pathological changes that the tissue undergoes from the in vivo position during radiological image acquisition (such as MRI or x‐ray) to the ex vivo histological sample that is examined under the microscope. The various types of deformations that occur include: in vivo tissue deformations from the preoperative image acquisition to the operating table, excision during surgery, slicing into typically 4–15 mm slabs, formalin fixation, sampling, dehydration, paraffin embedding, sectioning with the microtome to generate a thin histological slide typically 4–5 μm thick and rehydration for staining. Inevitably when a specimen is sliced into slabs the 3D structural information of the tissue is lost. The work described in this paper is primarily focused on reconstructing a 3D whole specimen volume from a fresh, sliced breast mastectomy sample. This is a vital component of the pipeline to establish correspondence between histopathology and in vivo imaging. We propose a novel 3D volume reconstruction algorithm and we demonstrate its use to map histology images to whole specimen radiological images (MRI and CT). The same methodology is also, in principle, applicable to other organs.

Reconstructing a 3D volume from images of a sliced specimen has been an active research field, but the primary focus to date has concerned organs that naturally undergo less severe deformations than the breast, such as the brain and the prostate. Often the goal of 3D reconstruction techniques has been the reconstruction of volumes from histological slices (typically around 4 μm thick) of tissue that has already been embedded in paraffin blocks. In preclinical small animal studies, 2D histological sections or autoradiographs have been used to reconstruct a 3D volume of a whole organ (in most cases the brain).^2^, ^3^, ^4^, ^5^, ^6^, ^7^, ^8^, ^9^ In some studies, this volume was subsequently used as a means of aligning histology to in vivo MRI,^10^, ^11^, ^12^ often using an additional image of the specimen before sectioning: either a specimen MRI^13^ or block‐face photographs of the paraffin block.^11^, ^12^, ^13^

In the above techniques, a 2D intensity‐based registration method was often employed, where one slice in the volume/stack was initially chosen as the reference image — this was usually in the center of the stack — and all the remaining images were mapped to the reference, using pairwise registrations between adjacent slices. Following this approach, Alic et al.^12^ used a rigid‐body transformation for alignment. Ourselin et al.^2^ proposed a rigid block‐matching transformation instead, where each slice was transformed with a single rigid‐body transformation that was calculated based on the local similarity of multiple patches/blocks between the images, rather than the global similarity across the entire images. Pitiot et al.^6^ used an alternative method, where the applied transformation was only locally rigid, within a circular neighborhood in the image. Finally, a block‐matching^10^ and a piecewise rigid transformation^11^ was used to align histology images to block‐face photographs. In these cases, there was no need for a 3D volume reconstruction, as the photographs were acquired before sectioning and therefore simply stacking them provided a coherent 3D volume of the brain.

Using pairwise registrations for the 3D volume reconstruction has two main disadvantages: it introduces a potential bias on the reference slice selection and it can result in noncoherent reconstructions, as each slice is transformed according to its similarity with only one neighboring slice. If one of these registrations fails, for example, due to a tear that occurred during sectioning, then all subsequent slices toward the end of the stack will also be misregistered. To address these problems, there have been various methods that proposed using more than one neighboring slice. Bagci et al.^7^ proposed the rigid pairwise alignment of separate subvolumes in the stack, which were then combined to provide the full volume. Yushkevich et al.^4^ have used multiple pairwise rigid registrations between each slice and a number of their neighbors in both directions in the stack. Then, they identified the path that consisted of the most successful registrations in order to connect neighboring slices and concatenated the transformations along that path. This way two neighboring slices could be aligned via one or more slices in the local neighborhood. Nikou et al.^3^ considered all slices in a local neighborhood of the stack simultaneously when transforming each slice, so that the similarity was computed between more than two images at the same time. A simultaneous alignment of each slice to all neighbors was also proposed by Feuerstein et al.,^9^ where a Markov random field formulation was employed for the optimization of the transformation parameters. Motivated by the same principle of providing more coherent and smooth volumes across slices, Cifor et al.^8^ have segmented brain images into gray and white matter and applied displacements on the contours of the slices, in order to produce smooth boundaries.

For human organ studies, existing approaches have been chiefly developed for prostate^14^, ^15^, ^16^, ^17^ and brain data.^18^, ^19^ Prostate studies have mainly focused on matching a single whole‐mount histology slide, or four normal size quadrants of the same plane to the in vivo MRI of the patient, without the need to reconstruct a 3D volume from serial slices. The proposed methodologies often require either manual interaction^16^, ^17^ or the acquisition of additional images of the whole ex vivo specimen before cutting and further slicing with the microtome. These additional images comprise a specimen MRI^12^, ^15^ or block‐face photographs of the sectioning process.^11^, ^15^ The use of an adapted specimen handling protocol involving 3D‐printed patient‐specific molds with cutting slots that allow even and parallel slicing of the specimen has also been proposed to facilitate alignment.^20^ Xiao et al.^21^ proposed a series of 2D and 3D affine registrations, where multiple sparsely sampled (i.e., unevenly spaced) histology sections were aligned simultaneously to an in vivo MRI. This produced a 3D histology pseudo‐volume, where the limited number of histology slides were interlaced with blank, zero‐value slices. In human brain studies, the acquisition of an ex vivo MRI of the specimen was proposed to facilitate the alignment: sparsely sectioned histology slides can then be registered in 2D to their corresponding MRI slices.^18^ The ex vivo MRI can then in turn be mapped to the in vivo MRI of the patient.^19^

The breast is a highly deformable organ and therefore there have been few attempts toward aligning in vivo to specimen images. In the most related work,^22^ single pathology slides from two patients were warped to ultrasound (US) images based on manually defined landmarks on the boundaries of a tumor, with a goal of facilitating the interpretation of US elastography images. Regarding 3D volume reconstruction, in preclinical research mammary glands of mice have been reconstructed either using rigid and elastic pairwise registrations between histology slides,^23^ or using block‐face imaging of the sectioning process and subsequent 2D alignment of each histology section to the corresponding block‐face image via a similarity transformation.^24^

In clinical breast studies, a 3D volume reconstruction, again from histology images, was proposed using various alignment techniques: a combination of manual interaction and affine^25^, ^26^ or pairwise B‐splines registrations,^27^ a semiautomated software package (FiAlign)^28^ and a pairwise rigid block‐matching approach.^29^ The motivation behind 3D histology volume reconstruction of a breast tissue block varied from providing an accurate measurement of tumor volume^25^, ^26^ to estimating the optimal sampling spacing between histopathology slides^29^ or facilitating the study of different DCIS^27^ and invasive carcinoma cases.^28^

**Figure 1:**
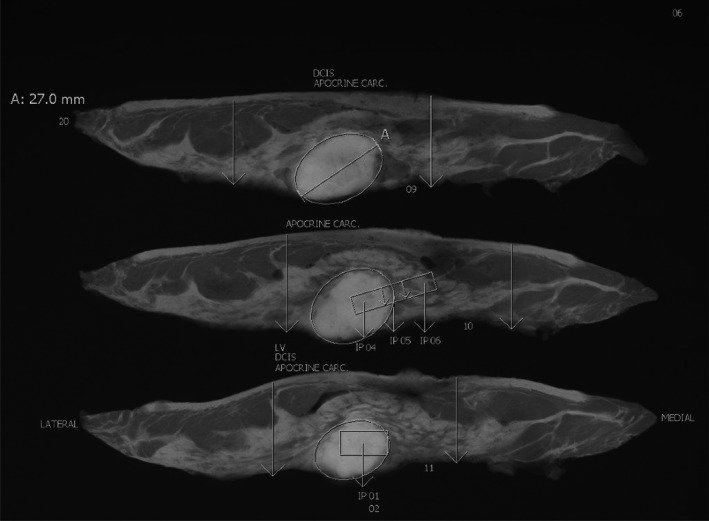
An example of the pathologist's annotations on the specimen radiographs, where the sampling position corresponding to the block that will produce a histology slide is indicated as the area that is in‐between the two vertical arrows. In this case, there were three large‐format histology slides generated with IDs: 09, 10, and 11. Each slab can generate zero, one, or multiple slides.

Typically after breast lumpectomy or mastectomy, the specimen is sliced into slabs, fixed in formalin, sampled, embedded in paraffin, and sliced with the microtome. In this study, we propose a novel technique to reconstruct a whole specimen volume from 2D radiographs of the specimen slabs. Although the specimen slicing protocol may vary between clinical sites (e.g., the slicing orientation can be axial, sagittal, or coronal, and the slab thickness can be typically from 4 to 15 mm), some imaging of the slabs is often acquired. The images can be either optical photographs or digital radiographs. The advantage of acquiring x‐ray radiographs is that the whole slab can be examined (rather than only its surface), avoiding reflection artifacts often present in optical photographs, providing better contrast and most importantly revealing information on the entire volume that otherwise would be obscured, for example, the glandular structure and the presence of microcalcifications and spiculations. The imaging of the slabs is used to indicate the positions where histology slides originate and allows pathologists to go back to the specimen for further sampling if required (as explained in detail in Section 2.A and shown in Fig. 1). We have previously presented preliminary results from our work in reconstructing a 3D whole specimen volume from 2D specimen radiographs of 4 mm thick fresh slabs.^30^, ^31^ The ultimate goal of this approach is to facilitate the alignment between histology and preoperative radiological imaging. Acquiring radiographs of the specimen slabs provides imaging information of the whole specimen rather than a smaller region of interest. The advantage of our method therefore is that individual histopathology slides can potentially be related back to in vivo imaging, via the whole specimen reconstruction, without the additional time and expense of reconstructing a 3D histology volume from serial 2D histological slides.

There are two main contributions of the work presented here. Firstly, the algorithm used for the 3D volume reconstruction provides a combination of two previously proposed techniques^2^, ^3^ and further improves the results by incorporating free‐form deformations (FFD)^32^ that allow nonrigid transformation of the slabs. The combination of neighborhood slice information with FFDs enables a more flexible, nonlinear deformation to be computed within the constraint that a coherent 3D specimen volume reconstruction is obtained. This is a critical refinement, given the highly deformable nature of breast tissue. We demonstrate the benefit of combining and extending these techniques on 10 clinical cases and provide quantitative evaluation. Secondly, this work provides the first attempt to date to reconstruct a 3D breast specimen volume from serial slab radiographs. We demonstrate how the reconstructed volumes can be used as an intermediate step in order to map histology slides from five clinical cases to whole specimen radiological images (MRI or CT) of the corresponding mastectomy samples.

## 2 Materials and methods

### 2.A. Materials

The specimen handling protocol after surgery typically follows the workflow briefly mentioned above: slicing into slabs, x‐ray imaging, formalin fixation, sampling, paraffin embedding, sectioning with the microtome, and staining. However, the workflow details at each stage can vary between clinical sites. For example, the slicing can be performed at different orientations, the thickness of the slabs can vary, and an x‐ray image or a photograph of the specimen can either be acquired at a different stage in the pipeline or not acquired at all. To gain a better understanding of the goal of this study, we describe below the data used in this work.

All images used in the study are mastectomy samples that were acquired at the Radboud University Medical Centre. As part of the clinical routine, the specimen handling at this site is as follows: initially the surgeon marks the specimen orientation using sutures and then the excised specimen is transferred to the pathology department, where it is inked, vacuum‐packed, and refrigerated to better preserve the tissue and also stiffen it to facilitate slicing. Then, the specimen is sliced axially using a meat slicing machine into 4–5 mm thick slabs. Using this method, instead of manual slicing, provides a standardization of the slicing process and ensures that all slabs have similar thickness and are parallel. Digital x‐ray images are then acquired using the hospital's x‐ray mammography system, with a typical image containing 2–6 slabs, depending on their size. The tissue is later fixed in formalin, sampled, put into cassettes and further processed into paraffin blocks. The approximate positions of the tissue samples selected for subsequent processing and staining, are annotated on the digital x‐ray images of the corresponding slabs. An example of these annotations is shown in Fig. 1. Details of the complete protocol can be found in.^33^

The goal of this work is to produce a 3D volume reconstruction from the x‐ray images of the specimen slabs that are acquired as part of the routine clinical practice. In this study, images from ten patients were used for validation. For five of these cases (p1–p5), there was one additional image acquired: a whole specimen MRI for one case, and a specimen CT for the remaining four as it was concluded that a specimen CT was quicker and more practical to acquire than MRI. This volume scan was acquired for research purposes before slicing, to validate the reconstruction algorithm and demonstrate the registration pipeline from the histology images to a whole specimen image of the patient. As the breast tissue is naturally highly deformable, the shape of the structures in the reconstructed volume can vary when compared to the whole specimen image. To account for this variation, the whole specimen MRI/CT of each patient was registered to the reconstructed specimen volumes. The transformation model used in all cases was initially a 3D rigid block‐matching, to recover the global transformation, followed by a fast implementation^34^ of the 3D FFD algorithm.^32^

The pixel size of all radiographs is [0.094 × 0.094] mm^2^ and the slab thickness is approximately 4 mm. The number of slabs in each mastectomy varies from 29 to 67. For the whole specimen images, the voxel size varied slightly. For the MRI of p1: [0.54 × 0.49 × 0.49] mm^3^, and for the CT of p2: [0.6 × 0.3 × 0.6] mm^3^, p3: [0.5 × 0.5 × 0.8] mm^3^, p4: [1.0 × 0.43 × 0.43] mm^3^, and p5: [0.92 × 0.92 × 1] mm^3^. All images were acquired at clinical scanners: The mammography system is a GE Medical Systems Senograph 2000D, the CT scanner is a Toshiba Aquilion ONE, and the MRI scanner is a 3T Siemens TrioTim. For the MRI, we used the T1‐weighted image for validation.

### 2.B. Methods

**Figure 2:**
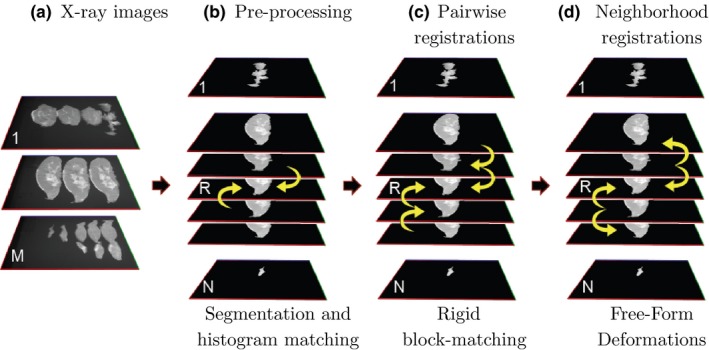
Overview of the proposed 3D reconstruction pipeline. The specimen slices are originally spread across M x‐ray images (a). During the preprocessing step, the slices are segmented to N individual images using connected components and the intensities are normalized to a reference slice R using histogram matching (b). The individual slices are first aligned using pairwise registrations (c). In this step, slice R in the middle of the stack is used as a reference image and remains unchanged. As we move toward the two ends of the stack, the remaining slices are registered to their single neighboring slice using a rigid block‐matching transformation. Finally, in a second registration task, each slice is transformed using FFD, considering the similarity to both of its neighboring slices to enforce structural coherence across slices (d). [Colour figure can be viewed at http://wileyonlinelibrary.com]

An overview of the pipeline is shown in Fig. 2. In this section, we use the more general term “slice”, rather than “slab” that specifically refers to thick slices, as the same methodology is applicable to the reconstruction of any tissue volume, from different type of slices. In this study, all slices are 2D radiographs of mastectomy slabs. The original radiographs typically contain more than one slice (Fig. 2(a)). In the preprocessing step, these are segmented into individual images and the intensities across slices are normalized (Section 2.B.1). The 3D volume reconstruction is completed in two steps: pairwise (Section 2.B.2.a) and neighborhood (Section 2.B.2.b) registrations.

#### 2.B.1. Preprocessing

As shown in Fig. 2(a), the slices obtained from a given specimen appear in sequence, in a number of x‐ray images, with each image typically containing 2–6 slices. Before registration, these are segmented from the background using a connected components algorithm. Manual interaction is only required for cases where the slices are in contact, with no clear boundary between them. A histogram matching technique is used for intensity normalization of the segmented slices, as intensity ranges vary between different x‐ray acquisitions. For this task, the slice in the middle of the stack is used as a reference image. Finally, all images are translated on the *X*‐axis to the center of the images for initialization of the registration tasks that follow. When the pathologist places the slabs next to each other for imaging, their position on the *Y*‐axis indicates the approximate position of the slices in the whole specimen, which is particularly useful for slices toward the two ends of the stack, as they are smaller than their neighbors. To preserve this information, a translation on the *Y*‐axis was not performed for initialization (Fig. 2(b)).

#### 2.B.2. 3D volume reconstruction

##### Pairwise registrations

To reconstruct a 3D volume, the individual slices are initially registered using pairwise registrations. As shown in Fig. 2(c), the slice in the middle of the stack *I*_*r*_ is used as a reference image and remains unchanged. Then as we move toward the two ends of the stack, the remaining slices are registered to their single neighboring slice. For example, slice *I*_*r*−1_ is registered to slice *I*_*r*,_ slice *I*_*r*−2_ is registered to slice *I*_*r*−1_, etc.

For this task, we used an intensity‐based approach with a rigid‐body block‐matching transformation 2 that was first proposed for the registration of serial histological sections from animal brain data. The advantage of the block‐matching technique is that it only assumes local similarities between sequential slices, rather than assuming that the anatomy is related across the whole image. Local rigid transformations are initially computed across local areas (blocks) and the final transformation is estimated using the most closely matching block‐pairs.

In our experiments, we used an implementation with a multiresolution scheme consisting of six levels. As in the original reference,^2^ the similarity measure is the correlation coefficient and the final transformation is computed using the *L*_1_ estimator, rather than least squares regression.

##### Registrations in a local neighborhood

Following the pairwise registrations with a rigid transformation, where the similarity is only computed between two images, we propose a subsequent registration step, where each slice is transformed according to its similarity to both neighboring slices (Fig. 2(d)). This approach was initially proposed for a 3D volume reconstruction from serial autoradiographic sections of a rat's brain.^3^ A key difference compared to the original method, and compared to the first stage of our 3D reconstruction, is the use of FFD instead of a rigid transformation. The combination of a nonrigid transformation model with the simultaneous alignment of each slice to its two neighbors favors coherence of structures across slices.

Each slice *I*_*i*_ is simultaneously aligned to both neighboring images *I*_*i *− 1_ and *I*_*i *+ 1_. As previously, the slice in the middle of the stack *I_r_* is used as a reference image and therefore remains unchanged and is not being transformed. For *N* slices, the parameters that are estimated are:

(1) $$\Phi =\{\Phi_{1},\ldots ,\Phi_{r-1},\Phi_{r+1},\ldots ,\Phi_{N}\},$$

where *I*_*r*_ is the reference image and Φ_i_ are the transformation parameters for each slice. The transformation parameters of the FFD in 2D are the x and y displacements of the control points in the mesh. Φ_*i*_ then denotes the $n_{x}^{i}\times n_{y}^{i}$ mesh of control points $\phi_{j,k}^{i}$ defined on image *I*_*i*_. The FFD can be written as:

(2) $$T_{\Phi_{i}}\left(p\right)=\sum_{m=0}^{3}\sum_{n=0}^{3}B_{m}\left(u\right)B_{n}\left(v\right)\phi_{j+m,k+n}^{i},$$

where *p = (x, y), j = ⌊x/n_x_⌋ − *1, *k = ⌊y/n_y_⌋ − *1, *u = x/n_x_ − ⌊x/n_x_⌋, v = y/n_y_ − ⌊y/n_y_⌋* and *B_m_* is the *m*‐th basis function of the B‐splines.

Considering the similarity, *S*, across all slices in the stack, the optimization problem of the global energy function *E*(Φ) can be defined as:

(3) $$\hat{\Phi }=\underset{\Phi }{\text{argmax}}\left(E\left(\Phi \right)\right),$$

where

(4) $$\begin{array}{cc} E(\Phi ) & =\sum_{i=1}^{N-1}E_{i}\left(\Phi_{i}\right)\\ & =\sum_{i=1}^{N-1}\sum_{j\in R_{i}}\sum_{p\in \omega }S\left(I_{i}\left(T_{\Phi_{i}}\left(p\right)\right),I_{i}\left(I_{\Phi_{i}}\left(p\right)\right)\right) \end{array}$$

where *R*_*i*_ is the neighborhood of image *I*_*i*_, or in other words its adjacent slices, and *I*_*i*_(*T*_Φ*i*_ (*p*)) is the image *I*_*i*_ at the transformed position *T*_Φ__*i*_ (*p*) using the parameters Φ_*i*_. Instead of optimizing the global energy directly across all images, the local energy *E*_*i*_ is optimized sequentially for all the slices, as in Ref.^3^. We used two neighboring slices in our implementation [*R*_*i*_ = (*i* − 1, *i* + 1)], as their thickness is 4–5 mm. This significant slice thickness means that more distant slices may bear little resemblance to the slice being registered and hence offer little or no benefit to the registration.

The control point grid that we used for this registration step is 8 × 8, resulting in 128 degrees of freedom. This choice was proven to be suitable for our digital radiographs dataset, as it provided adequate flexibility of the deformations, without resulting in any visible interpolation artifacts, which can occur in nonphysically realistic deformations. The similarity measure used is normalized cross‐correlation and the optimization scheme is gradient descent.

Our current implementation requires approximately 11 min for each registration task of one 2D slice, on a single core, 64‐bit machine, with a 2.8 GHz processor. The performance could be further optimized using a multithreaded implementation of the algorithm. The run‐time of the pairwise block‐matching implementation, used for the pairwise registrations,^35^ was 5 s for each 2D registration task on an eight‐core processor.

## 3 Results

### 3.A. Validation of the 3D volume reconstruction

To validate the quality of the reconstructed volumes, we present two sets of experiments. In Section 3.A.1, we assess the smoothness of the volumes that were reconstructed using specimen radiographs from ten clinical cases. For five of these cases, we used a whole specimen image (one MRI and four CTs) as a gold standard of the mastectomy samples in 3D. In Section 3.A.2, the reconstructed volumes are compared quantitatively and qualitatively to the whole specimen images.

#### 3.A.1. Assessing the smoothness of the reconstructed volume

In these experiments, we have used specimen radiographs from 10 clinical cases. To assess the smoothness of the reconstructed volumes in the direction of slicing, first we compute the distance of each slab contour (i.e., the outer boundary) from its two neighbors. In other words, for each slab *i* this distance is given by equation:

(5) $$d_{i}=\frac{d_{i,i-1}+d_{i,i+1}}{2},$$

where *d*_*i,j*_ is the mean Euclidean distance between the points in the contour corresponding to slab *i* and the closest points in the contour of slab *j*. The mean distance for all *N* slabs in each reconstructed volume is simply:

(6) $$d=\frac{1}{N-2}\sum_{i=2}^{N-1}d_{i}.$$

For each slab, Eq. (5) provides a distance metric of each slab contour from its two adjacent slabs (top and bottom) with a global minimum at the position that corresponds to the smoothest transition between the three slabs (i.e., the smoothest outer surface). Therefore, the mean of the distances for all slabs in the volume given from Eq. (6) should be smaller for the smoothest surface, although we do not expect this to be zero, as the slabs are not identical. The above distance metric provides a metric of surface smoothness, it has the advantage of being independent of the slice thickness (as it operates in 2D) and it works directly on the image intensities, rather than surrogate triangulated meshes. The first and last slab in the stack are excluded from the calculation as they only have one adjacent slab.

For every patient, we have computed the metric given by Eq. (6) for each volume that is reconstructed using the three following techniques:

1. The original position of the slabs before registration, where a translation across the *X*‐axis was used according to the slabs’ center of mass (X‐TR volume).
2. A pairwise rigid block‐matching algorithm^2^ (P‐BM volume).
3. Our proposed reconstruction method with FFD and simultaneous registration of each slab to its two neighbors (FFD‐N2 volume).

**Figure 3:**
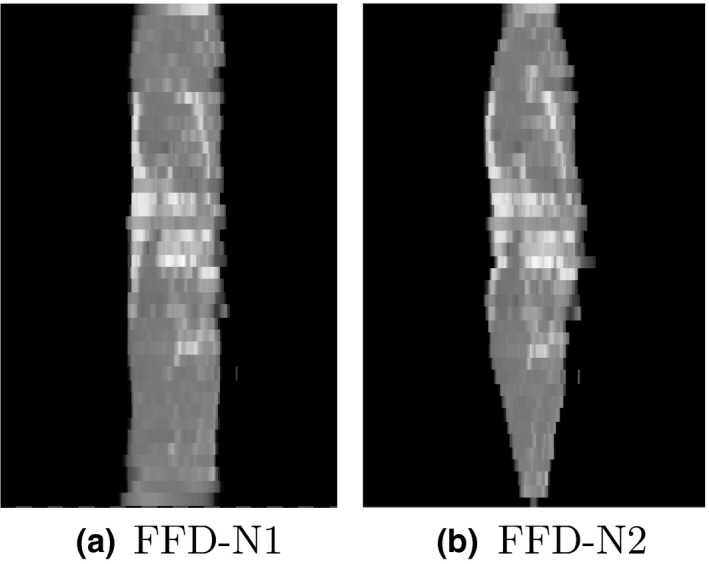
Comparison of a volume reconstructed from the same 2D radiographs of p6 using (a) FFD and pairwise registrations — i.e., one neighboring slab (FFD‐N1) — and (b) FFD and two neighboring slabs (FFD‐N2). This is a sagittal view for a volume reconstructed from axial specimen slabs.

It is worth noting that comparing our approach against a pairwise FFD registration between slabs would not provide a meaningful comparison, as all slabs would be stretched to match the area corresponding to the reference image. As illustrated in Fig. 3, this approach produces nonphysically realistic deformations of the slabs.

**Figure 4:**
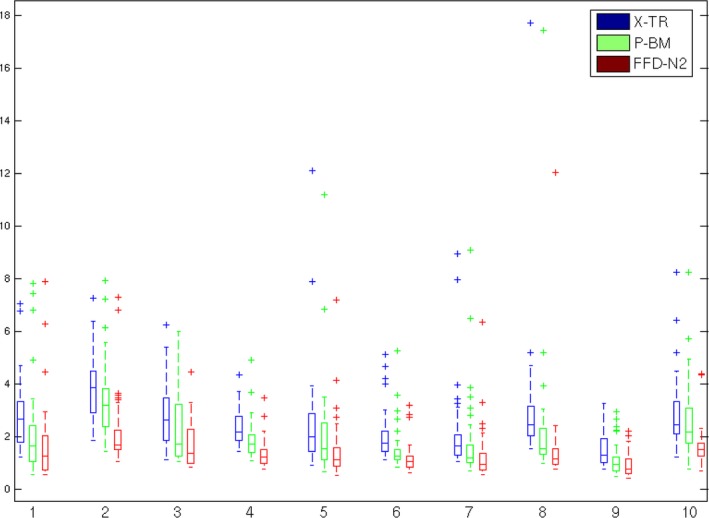
Boxplot of the contour distances given by Eq. (6) for each of the three volume reconstruction techniques. The X‐axis corresponds to the patient number and the Y‐axis to the contour distances in mm. [Colour figure can be viewed at http://wileyonlinelibrary.com]

A boxplot of the contour distances, given by Eq. (6), is shown in Fig. 4. The plot illustrates that our proposed method provides a clear improvement compared to the X‐TR volume and the P‐BM technique. In all cases, the mean, standard deviation, maximum and minimum distance values are lower for our approach. A paired t‐test showed that the results of FFD‐N2 were statistically significantly different both from X‐TR (*p* = 1.0966 · 10^−6^ with 9 degrees of freedom) and from P‐BM (*p* = 1.192 · 10^−4^ with 9 degrees of freedom). In all the paired t‐tests performed, we have used 0.05 as a significance level of the null hypothesis rejection.

**Figure 5:**
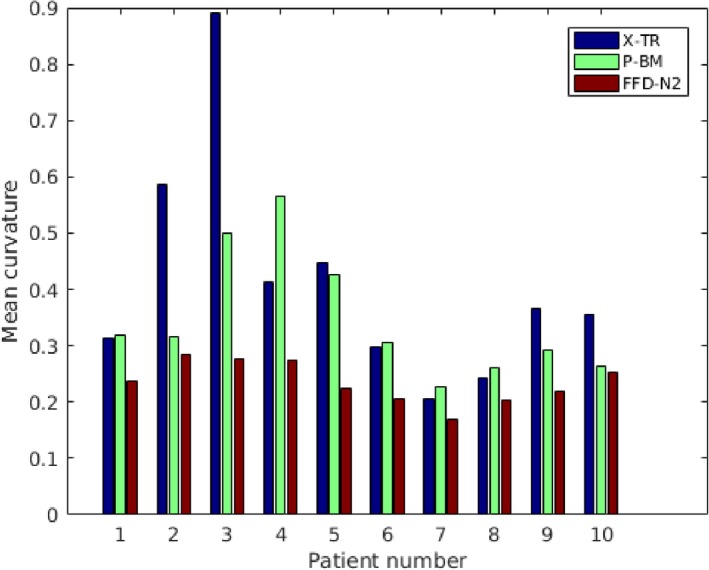
Plot of the mean curvatures computed from the surface of each volume reconstruction technique. The X‐axis corresponds to the patient number and the Y‐axis to the average absolute value of the mean curvatures across the surface. [Colour figure can be viewed at http://wileyonlinelibrary.com]

An alternative surface smoothness measure can be provided by the mean curvature computed from the surfaces that are extracted from the reconstructed volumes, for example, using the marching cubes algorithm. Figure 5 shows the mean curvatures for each of the 10 volumes computed as the average of the absolute values of the mean curvatures across the whole surface, as we are not interested in the variation between positive and negative curvatures. In all cases, the average values are lower for our approach. A paired t‐test showed a statistically significant difference between the results of FFD‐N2 and X‐TR (*p* = 0.0106 with 9 degrees of freedom) and FFD‐N2 and P‐BM (*p* = 0.004 with 9 degrees of freedom).

**Figure 6:**
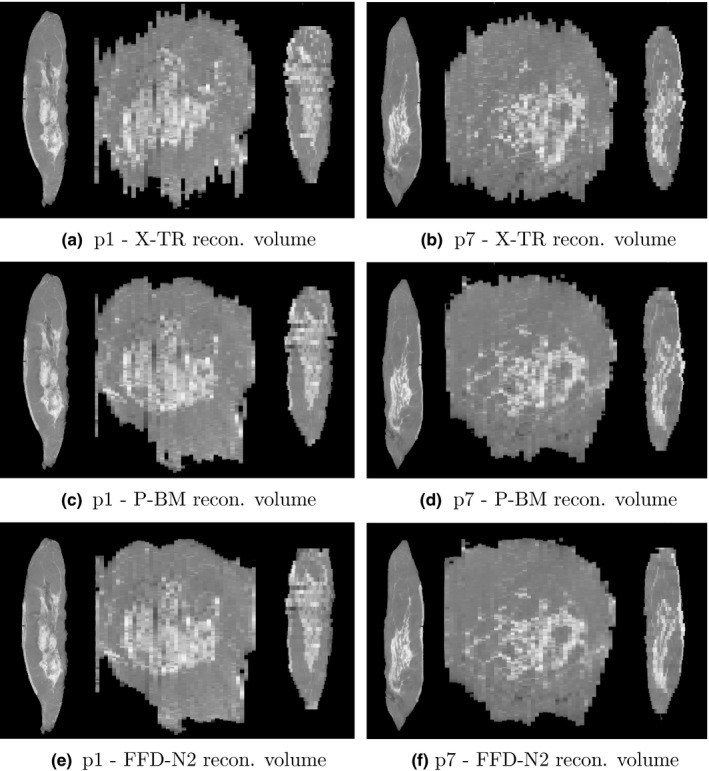
Reconstructed volumes for p1 and p7 using three different methods. From left to right in each image: axial, coronal, and sagittal planes.

**Figure 7:**
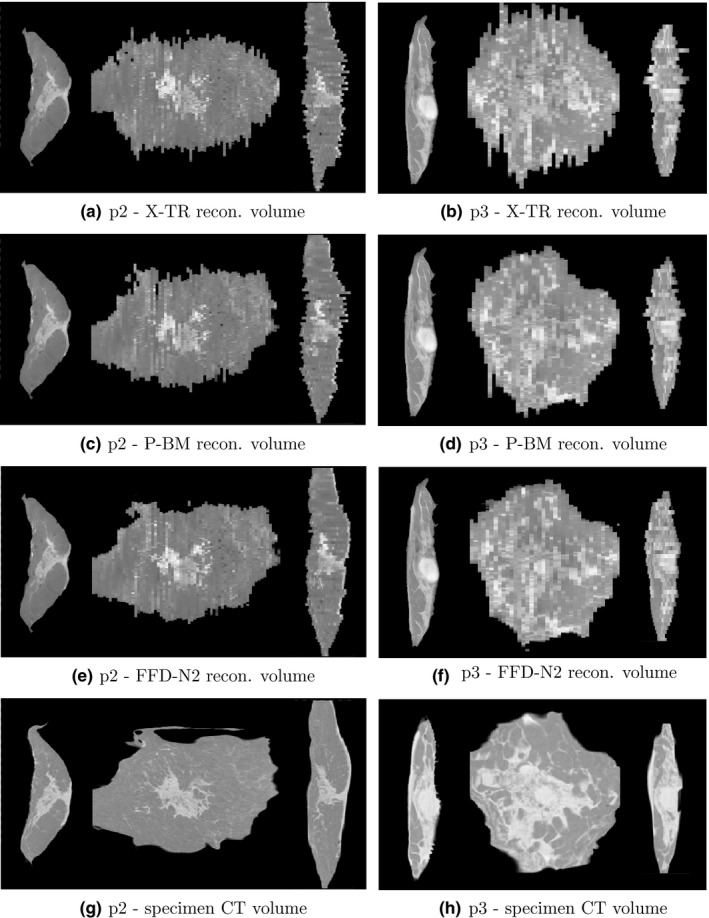
Reconstructed volumes for p2 and p3 using three different methods (a)–(c) and the whole specimen CT registered to the FFD‐N2 volume. From left to right in each image: sagittal, coronal, and axial planes.

Some examples of the reconstructed volumes computed with all three methods mentioned above are given in Figs. 6 and 7(a)–(f). A visual comparison between the three sets of coronal and sagittal views shows the benefit of using a flexible transformation model in combination with a neighborhood information between the slabs.

#### 3.A.2. Validation using a whole specimen image

The above experiments provide a quantitative evaluation of the volume smoothness when looking at the outer surface of the reconstructions. For the assessment of the internal breast structures, we propose the use of a whole specimen image that is acquired before slicing, as the gold standard of the fibroglandular structures’ appearance inside the breast. These images are used to validate the proposed 3D volume reconstruction method and are not routinely acquired in clinical practice. For this task, we used a subset of the above patients (p1–p5). As described in Section 2.A, the whole specimen MRI/CT of each patient was registered to the three reconstructed specimen volumes (X‐TR, P‐BM, and FFD‐N2) using three separate registration tasks.

Two examples of these registration tasks are shown in Fig. 7. Spatially corresponding structures between the two images can be seen for both patients. Patient p2 (Fig. 7) is particularly challenging, due to the large volume of the mastectomy sample. This volume consists of 67 slabs, while all remaining nine cases in our study consist of 29–49. Also, due to the difficulty in slicing this sample with the machine, the slicing was performed sagittaly, rather than axially which is typically performed for all cases.

##### Assessment based on image similarity

**Figure 8:**
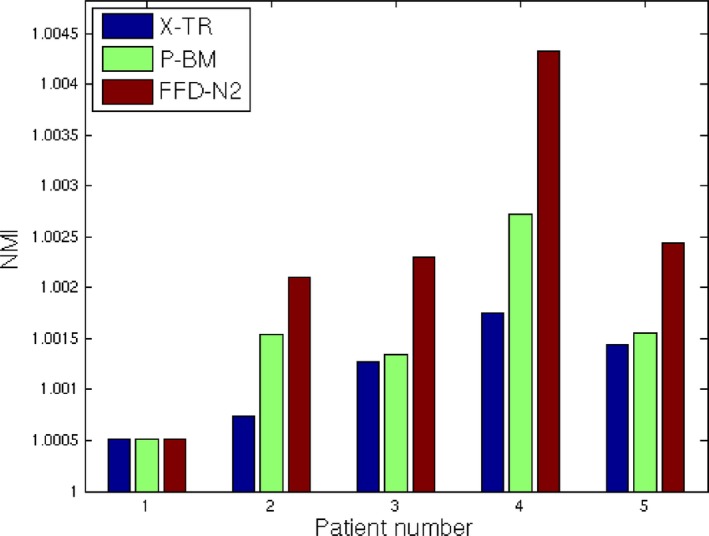
NMI value between the specimen volume (MRI for p1 and CT for p2–p5) and the reconstructed volume with each one of the three methods — X‐TR, P‐BM, and FFD‐N2. In all three cases, the specimen volume is registered to the reconstructed volume following separate 3D registrations. [Colour figure can be viewed at http://wileyonlinelibrary.com]

To quantify the similarity between the 3D images, we have computed the normalized mutual information (NMI) similarity measure between the whole specimen volume (ex vivo MRI or CT) and the volumes that were reconstructed from the specimen radiographs following each of the three methods: X‐TR, P‐BM, and FFD‐N2. This value provides a quantitative measure of how well the internal fibroglandular structures in the reconstructed volume match the specimen volume before slicing, as all the intensities in both images are considered in the computation. The results are illustrated in Fig. 8. We can see that for four of five cases our proposed method provides an improvement over X‐TR and P‐BM; for p1 all three methods produce similar results. For these experiments, we used an ITK^36^ implementation of the NMI as introduced by.^37^

##### Error estimation using point correspondences

**Figure 9:**
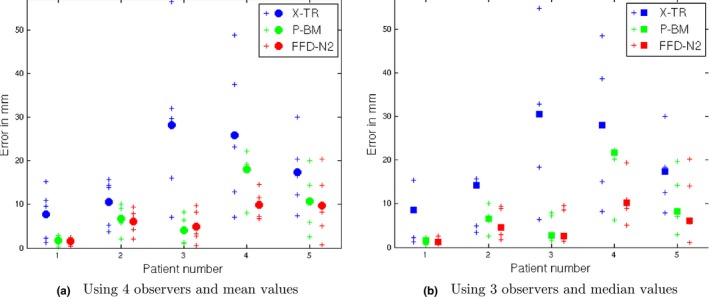
TREs of the three reconstruction methods using manual annotations as the gold standard correspondence. (a) Results from all four observers, where the errors are calculated using the mean TRE per patient. The crosses (+) correspond to the five individual point errors and the circles (•) to the mean values computed from five points per patient. (b) Results from three observers (the observer furthest from the mean of the other three observers was discarded), where the TRE is computed using the median, rather than the mean value. The crosses (+) correspond to the five individual point errors and the squares (□) to the median values computed from five points per patient. [Colour figure can be viewed at http://wileyonlinelibrary.com]

**Figure 10:**
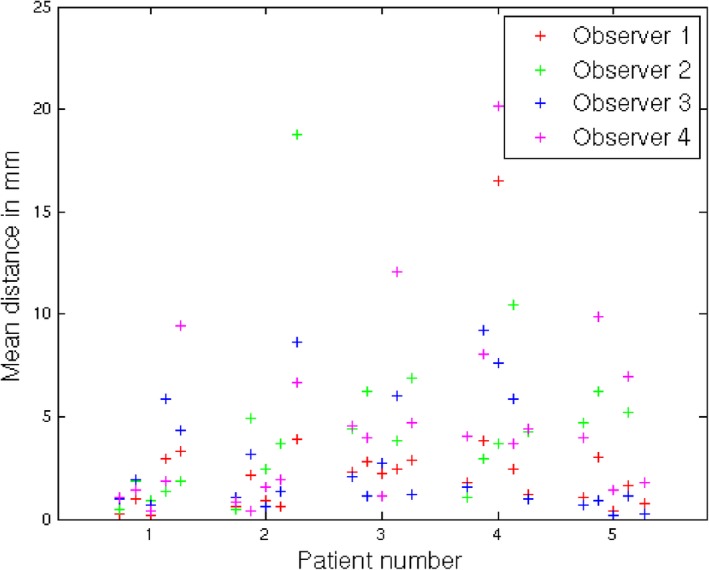
Interobserver variability results for the observer study. There were five points annotated per patient (corresponding to the five columns per patient in the plot). All points were annotated by four observers, illustrated by different colors. Each cross corresponds to one point/landmark and indicates the distance of this observer's annotation from the coordinate that is computed using the mean position of the other three observers’ annotation, i.e., the consensus. The influence of outliers in the gold standard computation was reduced when using the three most proximal observers for each point (the one furthest from the mean of the other three observers was discarded). [Colour figure can be viewed at http://wileyonlinelibrary.com]

Finally, to assess whether our volume reconstruction technique results in better target registration errors (TREs) when mapping points from the specimen radiographs to the whole specimen images (MRI or CT), we have used as a gold standard manually identified correspondences from four observers (medical imaging scientists). All observers were given the same five features per patient (25 points in total) on the original specimen radiographs in the X‐TR volume and were asked to manually identify the corresponding positions in the undeformed specimen volume (resulting in 100 annotations). The annotated landmarks were anatomical features (of either tumor positions or normal parenchyma) that could be identified in both the reconstructed volumes and the whole specimen images. The mean positions of the observers’ corresponding annotations in the whole specimen volume were used as the gold standard to compute the TREs for each one of the three volume reconstruction techniques. The results are shown in Table 1 and Figure 9(a). A paired t‐test showed a statistically significant difference between the results of FFD‐N2 and X‐TR (*p* = 1.5275·10^−4^ with 24 degrees of freedom) and FFD‐N2 and P‐BM (*p* = 0.0424 with 24 degrees of freedom). As the computation of the gold standard from manual annotations can be affected by outliers, we have also computed the error by generating the gold standard from the three most proximal observers for each feature point and discarding the fourth observer's annotation (i.e., the annotation which is furthest from the mean position of the other three observers). In this case, the TREs for all 25 points were computed using the median values, to account for the effect of outliers when computing the TREs. The results are shown in Table 1 and Figure 9(b). We can see that FFD‐N2 provided the lowest TREs, with a median error of 5 mm, when accounting for the effect of outliers. It is worth noting that the slab thickness of the radiographs is 4 mm and the interobserver variability is 3.4 mm. Figure 10 illustrates the interobserver variability for each point used for validation. The results of the TREs further confirm the visual observations and the assessment based on image similarity.

**Table 1:** TRE values for all 25 points using the annotations of all four observers and the mean values (left), and using three observers (the one furthest from the mean of the other three observers was discarded) and the median. All values are in mm

	Four observers		Three observers	
	Mean	Std	Median	Std
X‐TR	17.9	14.1	14.9	14.1
P‐BM	8.2	7.1	6.6	7.3
FFD‐N2	6.4	5	5	5.5

### 3.B. Dependency on the reference slab and the quality of the slabs

In the following experiments, we test the dependency of our proposed algorithm (a) on the chosen reference slab and (b) on the presence of artifacts in the slabs.

#### 3.B.1. Altering the reference slab

To assess the sensitivity of the proposed algorithm on the specific reference slab chosen, we have repeated the 3D volume reconstructions of patients p1–p5 four times, using each time as a reference the slab *r* ± 2 and *r* ± 1 instead of *r*, which is in the middle of the stack. In our experiments, the differences between the five reconstructions were visually minimal and were localized around a small number of slabs around the middle of the stack. The quantitative evaluation of the contour distances showed a mean difference in the overall distance measure *d* (across all slabs of 0.038 ± 0.176 mm. The mean difference in the TREs computed from the point correspondences (across all points and using all observers) was 0.03 ± 2.1 mm, resulting in mean TREs in the range of [6.3–6.7] mm (which is comparable to the mean of 6.4 mm computed for slab *r* and shown in Table 1). The choice of the reference slab is not expected to have a large effect on the final reconstruction, provided that the selected slab is toward the center of the stack and does not contain any large artifacts or significant deformations compared to the remaining slabs. In our dataset, there were no large artifacts in the slab radiographs used as reference.

#### 3.B.2. Simulating slab artifacts

**Figure 11:**
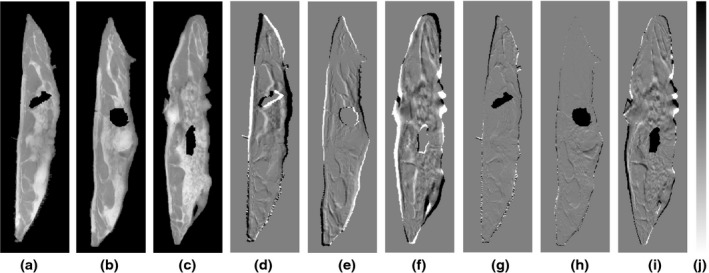
Illustration of the FFD‐N2 algorithm behavior with artificially inserted holes in the slabs of p3, (a)–(c). (d)–(f): Differences between the images before and after application of FFD‐N2 on the images with holes. (g)–(i): Differences between the images produced using the reconstruction with and without holes. (j) Gray bar corresponding to the difference between images (d)–(i): the range is from −4621 (top) to 4621 (bottom), which is the maximum intensity in the volume. The midpoint in the bar corresponds to zero difference in the intensities between the images.

In this work, we focus specifically on reconstructing a whole mastectomy sample from slab radiographs of a sliced specimen. As explained in Section 2.A, the specimen is sliced with a meat slicing machine into 4–5 mm thick slabs. Therefore, although there are clear deformations in each individual slab as shown in the images, these are less likely to have tears and holes, as they are relatively thick (e.g., compared to histology slices, whose thickness is around 4 μm). As in our dataset of 10 clinical cases, there were no large tears or holes in the specimen slabs, we have artificially inserted holes of various shapes and sizes on some of the specimen slabs of case p3 (shown in Fig. 11(a)–(c)) before running the FFD‐N2, to test the dependancy of our algorithm on the presence of holes. Figure 11(d)–(f) shows the effect of the FFD‐N2 on each slab, displaying the difference images before and after the application of the algorithm on the images with holes. We have compared the results with our original reconstruction without the holes (Fig. 7(f)) and found that the effect on the deformation of the three slabs was minimal. The difference images between the reconstructed slabs with and without the holes are shown in Fig. 11(g)–(i). The mean, standard deviation, minimum and maximum values of the contour distance metric remained the same across the volume when computed with and without the insertion of holes. In general, small tears and holes are expected to not significantly affect the performance of the FFD‐N2 algorithm, as the control point spacing is relatively large, favoring smoother deformations of the slabs and avoiding localized nonrealistic deformations.

### 3.C. Demonstration of the mapping between histology and whole specimen imaging

Finally, we demonstrate the potential of using our volume reconstruction method for mapping hematoxylin and eosin (H&E)^38^‐stained histology sections to a whole specimen radiological image for p1–p5. This is an important step in aligning histopathological to radiological images that can be further extended in future work to include in vivo radiological images, such as MRI or x‐ray mammograms. The pipeline of the image processing and registration tasks is:

1. Conversion of H&E sections to grayscale images.
2. 2D registration (rigid block‐matching followed by FFD) of each histology slide to the corresponding specimen radiograph of the slab from which the histology slide was originated. The manual annotations performed by the pathologists (Section 2.A) were used for initialization.
3. 3D specimen volume reconstruction from the 2D specimen radiographs using FFD‐N2.
4. 3D registration (rigid block‐matching followed by FFD) of the specimen MRI/CT to the reconstructed volume.

**Figure 12:**
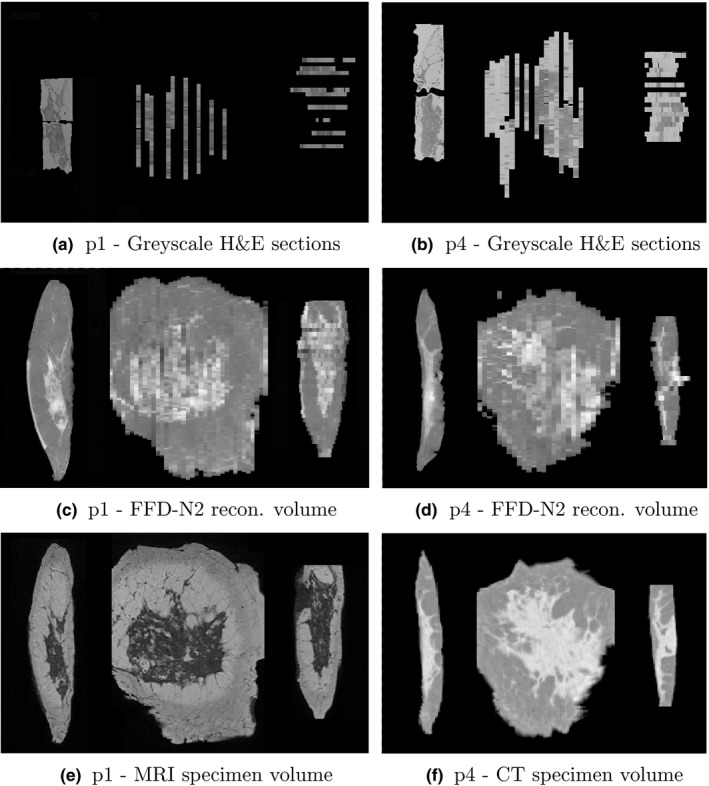
Mapping histology to a specimen MRI (CT) via the reconstructed volume for p1 (p4). From left to right in each image: axial, coronal, and sagittal planes.

**Figure 13:**
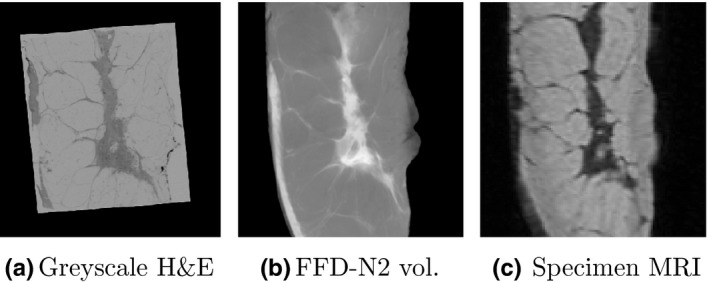
Mapping histology to a specimen MRI via the reconstructed volume for p1. All axial planes.

Figure 12 illustrates the results of the full pipeline from histology to specimen imaging for patients p1 and p4. The histology slides have been transformed using the concatenation of the two transformations from the steps (2) and (3) above. We can see that the H&E slides do not cover the whole mastectomy specimen, only the areas that were sampled by the pathologists. Also, although their thickness is approximately 4 μm, we do not know exactly at which depth of the corresponding 4 mm slab they originate, therefore Fig. 12 displays their thickness as 4 mm, to match the slab thickness. Figure 13 is an example of one histology slide, from subject p1, showing all three images aligned (H&E, specimen radiograph and MRI). In all cases, we can see correspondences between the images despite the differences in contrast and resolution.

## 4 Discussion

In the experiments described above, we first validated our proposed volume reconstruction technique using a distance metric between the contours of the 2D slabs. This provides an independent evaluation of the volume smoothness orthogonal to the direction of the slicing, as the 2D registrations between slabs were all performed using an intensity‐based technique. In the qualitative and quantitative results presented in Figs. 6, 7 and 4, we can see the gradual improvement of the volume smoothness as we move from the X‐TR volume, to P‐BM and finally to FFD‐N2. The improvement in smoothness is apparent in all four measures: mean, standard deviation, minimum and maximum value. Overall, our proposed method improves the mean distance between contours by 42% (mean: 1.5 ± 1.0 mm) compared to the X‐TR volume before registration (mean: 2.6 ± 1.4 mm) and by 28% compared to P‐ BM (mean: 2.1 ± 1.4 mm). These improved reconstruction results are also reflected in the overall mean surface curvature in Fig. 5 and in the visual assessment of the images from all patients.

The first set of experiments provides a quantitative evaluation of the volume smoothness when looking at the outer surface of the reconstructed volume. The assessment of the internal breast structures is more complicated. One could propose to segment the fibroglandular tissue from the specimen radiographs and apply the same evaluation metric that was applied to the outer contours above. However, due to the complexity of the breast fibroglandular tissue topology and the fact that the slabs are relatively thick and therefore there are often great differences even between adjacent slabs, this segmentation task does not guarantee the continuity of the segmented structures across slabs. Small isolated structures that appear in one slab might disappear in the next or they might become connected. The contour distances between slabs that are not composed of the same structures would therefore not provide meaningful results.

We propose instead the use of a whole specimen MRI or CT image as a gold standard. The benefit of using a flexible transformation for the reconstruction is particularly clear in the visual assessment of the results for p2, as the rigid transformation (Fig. 7(c)) cannot recover the large deformations that the tissue undergoes during slicing. Despite the large volume of the sample, we can see good correspondence between the FFD‐N2 volume and the specimen CT [Figs. 7(e) and 7(g)]. In addition to providing a gold standard for visual assessment, the specimen volumes can be used to perform quantitative validation, by computing the 3D similarity to the reconstructions, and the TREs based on observers’ manual annotations. For this task, the specimen volumes were all registered to the reconstructed volumes. The breast is a particularly deformable structure and therefore a 3D registration is essential for the spatial correspondences to become apparent. Even when using a standardized slicing protocol with a meat slicer, such as the one followed in our study, the breast tissue deformations introduced by slicing are significant. The final TREs computed using FFD‐N2 were in the order of 5–6 mm, which is close to the interobserver variability (3.4 mm) and the slab thickness (4 mm).

The proposed method can also be applied to data originating from other pathology centers that routinely acquire some imaging of the specimen slabs (either radiographs or photographs). It is also applicable to other datasets, for example, histology slides that are densely sampled (i.e., finely spaced sections). In this case, some of the parameters can be adapted to suit the specific application. For example, for thin histology slides, a larger number of neighboring slices could be used, as the coarse scale anatomy would not vary significantly between a few neighboring images. Using a larger number of neighbors would add further robustness to large tears and holes that can occur in histology slices and cannot be handled by pairwise registrations.

After validation of the 3D volume reconstruction algorithm, we have performed a set of experiments to illustrate that the algorithm is robust to the presence of holes in the slabs and that the exact reference slab choice does not have a large effect on the final reconstruction result, provided this is located approximately toward the center of the stack and it does not contain any large artifacts.

The last experiment demonstrates the potential of using the reconstructed volume to map H&E stained histology sections to a whole specimen radiological image (MRI or CT). Ultimately, the goal is to map the histology images to an in vivo image of the patient, such as the MRI, using the reconstructed volume from the specimen slab radiographs, without the need of the additional whole specimen image. Matching 2D histology slides that are 4 μm thick to a 3D breast volume can be very challenging, therefore providing some 3D position of the slides across the volume can help simplify this task. Reconstructing a 3D coherent specimen volume from the slab radiographs will allow a diffeomorphic transformation to be estimated from the specimen volume to the preoperative imaging. Naturally, the deformation from the in vivo prone position in the MR scanner to the ex vivo position of the specimen lying flat on the pathology bench is very complex. There are previously proposed techniques that focused on the in vivo prone to supine MRI alignment for surgical planning.^39^, ^40^, ^41^, ^42^ These could be extended to map the in vivo supine MRI to the specimen mastectomy volume.

Finally, an additional clinical application for the spatial correspondence between in vivo radiological and pathological imaging is its use as a tool to help pathologists decide which parts of the excised tissue are most critical to sample for further processing and staining. For example, a pathologist might want to sample a specific region that was enhancing on the preoperative MRI. This can be a challenging task, for example, for certain DCIS cases that are not palpable and certain lobular carcinomas that do not provide visible contrast in the specimen radiographs.

## 5 Conclusion

We have presented a method for the 3D reconstruction of a volume from serial breast specimen slab radiographs. The proposed technique provides further improvements compared to related existing techniques that were developed for brain data.^2^, ^3^ Our algorithm has the advantage of combining a flexible transformation model, with neighborhood slice information when deforming each of the slices in the volume, providing improved coherency of the structures across slices, which is essential for breast specimen samples. The transformation model that we use is FFD, but the algorithm can also be adapted to incorporate a different model. In our implementation, using FFD and deforming each slab to match both adjacent slabs provided adequate constraints, without the need for any additional regularization term commonly used in conjunction with FFD.

The proposed technique was applied to clinical mastectomy cases. Qualitative and quantitative assessment of the reconstructed volumes has illustrated the benefit of using this methodology, resulting in TREs of 5 mm. To our knowledge, this is the first method that has been applied to clinical breast cases, with the goal of reconstructing a whole specimen sample.

Initial experiments on mapping clinical H&E sections to whole specimen radiological imaging showed promising results. A future study could focus on the development of an algorithm that would allow the alignment of the specimen reconstructed volume to an in vivo patient image, such as MRI or x‐ray mammography, thus providing a complete pipeline from histology to in vivo radiological imaging.

## Conflicts of interest

The authors have no relevant conflicts of interest to disclose.
